# Influence of Temperature on FLD and CLD Damping Treatment

**DOI:** 10.3390/ma19173674

**Published:** 2026-08-29

**Authors:** Piotr Łabuński, Lucjan Witek, Paweł Obal

**Affiliations:** 1 Department of Applied Mechanics and Robotics, Rzeszów University of Technology, al. Powst. Warszawy 8, 35-959 Rzeszow, Poland; p.obal@prz.edu.pl; 2 Department of Aerospace Engineering, Rzeszów University of Technology, al. Powst. Warszawy 8, 35-959 Rzeszow, Poland; lwitek@prz.edu.pl

**Keywords:** damping materials, viscoelastic materials, butyl rubber, bituminous material, passive damping, influence of temperature, experimental modal test, free-layer damping (FLD), constrained-layer damping (CLD)

## Abstract

The damping capabilities of viscoelastic materials depend heavily on the operating temperature. Since these materials are used for passive vibration damping, it is necessary to determine the damping properties of selected materials across a range of temperatures. This paper presents the results of an experimental investigation into the effect of temperature on the damping performance of viscoelastic materials applied to aluminium cantilever beams using free-layer damping (FLD) and constrained-layer damping (CLD) techniques. Butyl rubber and bituminous materials were experimentally tested by modal analysis over a temperature range from 22 °C to −2 °C. Frequency response functions, resonance frequencies, resonance amplitudes, and modal loss factors were determined for selected bending modes. The results show that CLD provides better damping at room temperature, particularly for butyl rubber, due to enhanced shear deformation in the viscoelastic layer. However, as the temperature decreases, material stiffening alters the damping efficiency, and FLD becomes more competitive or superior in several modes. The findings confirm that passive damping effectiveness is strongly dependent on temperature, material type, and vibration mode.

## 1. Introduction

Mechanical vibrations arise from a wide variety of sources, including environmental excitation, as well as the operation of machines, engines, and rotating components. Once introduced into a structure, vibrational energy can be transmitted through its components and may contribute to both structural loading and noise generation. From the human perspective, prolonged exposure to vibration and noise may adversely affect comfort, well-being, and physical performance. From the structural perspective, cyclic loading associated with vibration increases stress fluctuations and may accelerate fatigue damage. Consequently, vibration and noise reduction remains an important consideration in the design of mechanical systems [1,2,3,4].

These issues are particularly relevant to aircraft, where crew members may be exposed to vibration for extended periods. Such exposure can increase fatigue and discomfort and may negatively affect the ability of pilots to perform demanding tasks. Previous investigations have also associated long-term vibration exposure with musculoskeletal problems, including neck and back disorders among aircrew [5,6]. For this reason, acceptable levels of whole-body vibration must be considered when assessing the working environment of aircraft occupants [7].

Vibration also influences the durability of aircraft structures. Periodic dynamic loads generate alternating stresses that contribute to fatigue accumulation. For example, excitation associated with engine rotor imbalance may simultaneously increase cabin noise and promote fatigue damage in highly loaded engine components, including compressor blades [8]. Effective vibration reduction is therefore important not only for improving comfort but also for maintaining structural integrity.

One widely used approach to passive vibration control consists of applying viscoelastic damping layers to vibrating structural surfaces. Surface damping solutions are attractive because they can provide considerable energy dissipation without the need for active control equipment and can usually be incorporated into a structure at relatively low cost [9]. Such treatments are used in both automotive and aerospace engineering [3,10]. Commercial damping mats are commonly manufactured from materials such as butyl rubber or bituminous compounds.

**Figure 1:**
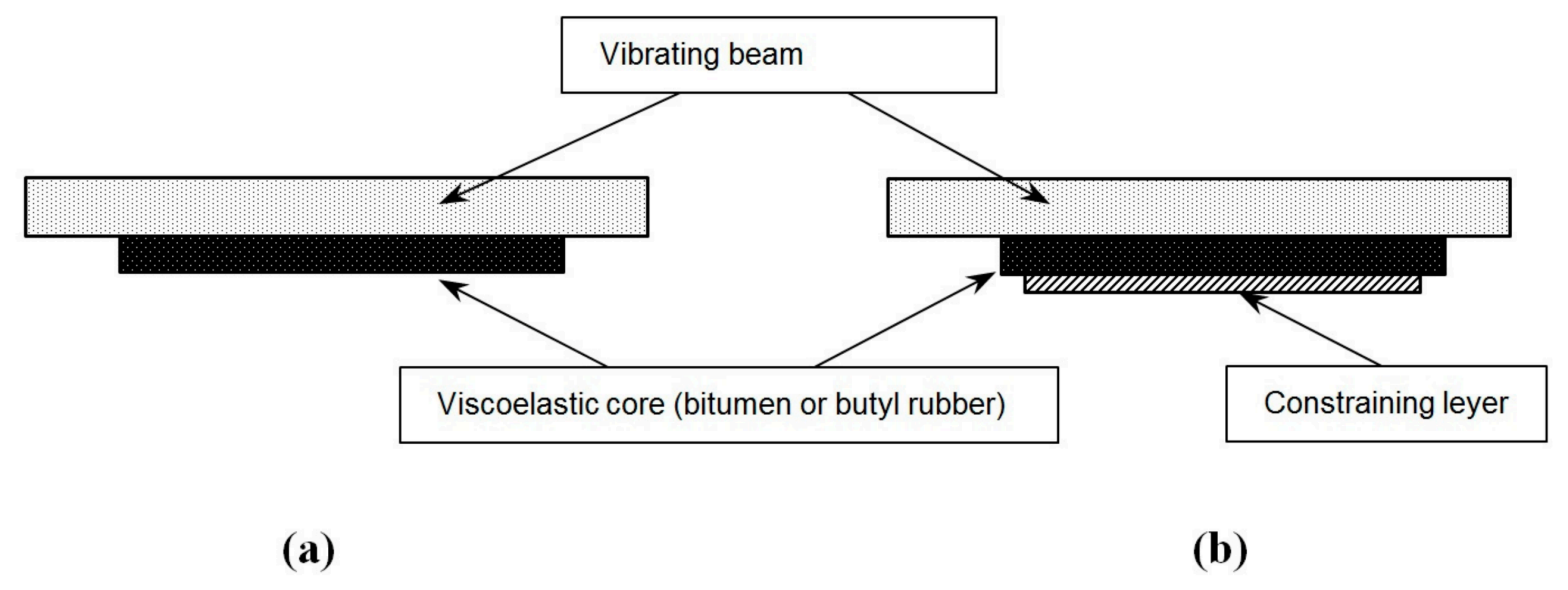
Viscoelastic damping materials in configuration: (**a**) FLD treatment and (**b**) CLD technique [17,18].

Two representative arrangements of viscoelastic surface damping are free-layer damping (FLD) and constrained-layer damping (CLD), schematically illustrated in Figure 1 [11]. In an FLD system, the viscoelastic layer is bonded directly to the surface of the vibrating structure. Structural bending causes deformation of this layer, through which part of the mechanical energy is dissipated. In a CLD system, an additional thin constraining sheet is bonded to the outer surface of the viscoelastic layer. The relative deformation of the base structure and the constraining layer produces shear deformation within the viscoelastic material, which constitutes an important mechanism of energy dissipation [12,13].

The performance of both configurations depends strongly on the mechanical properties of the viscoelastic layer. Since these properties vary with environmental and operating conditions, temperature may substantially change the damping effectiveness of a given treatment [14]. A commonly used parameter for quantifying structural damping is the loss factor, η [15,16]. Larger values of η indicate that a greater fraction of vibrational energy is dissipated during cyclic deformation.

Previous studies by the authors investigated the vibration-damping performance of selected viscoelastic materials and examined FLD and CLD treatments under room-temperature conditions [19,20]. Subsequent work considered the temperature dependence of butyl rubber and bituminous damping layers in an FLD configuration over the range from −2 °C to 22 °C [21]. These investigations demonstrated that the mechanical response of the damping layer changes noticeably with temperature and indicated the need for a direct comparison of FLD and CLD arrangements under the same thermal conditions.

Accordingly, the present study extends the previous investigations by experimentally comparing the temperature-dependent behaviour of FLD and CLD treatments. Two commercially available viscoelastic materials, namely butyl rubber and a bituminous material, are examined over the experimentally accessible temperature interval from −2 °C to 22 °C. The comparison focuses on changes in frequency response characteristics, resonance frequencies, resonance amplitudes, and modal loss factors. The study is intended to improve the understanding of how the damping mechanism associated with each configuration changes with temperature and to provide experimental evidence relevant to the selection of passive damping treatments for lightweight structural components.

## 2. Materials and Methods

The experimental procedure employed in the present study was based on the modal-testing methodology previously developed by the authors and described in [21]. The same principal measurement equipment and temperature-control system were used, while the present investigation was extended to enable a direct comparison between free-layer damping (FLD) and constrained-layer damping (CLD) configurations.

**Figure 2:**
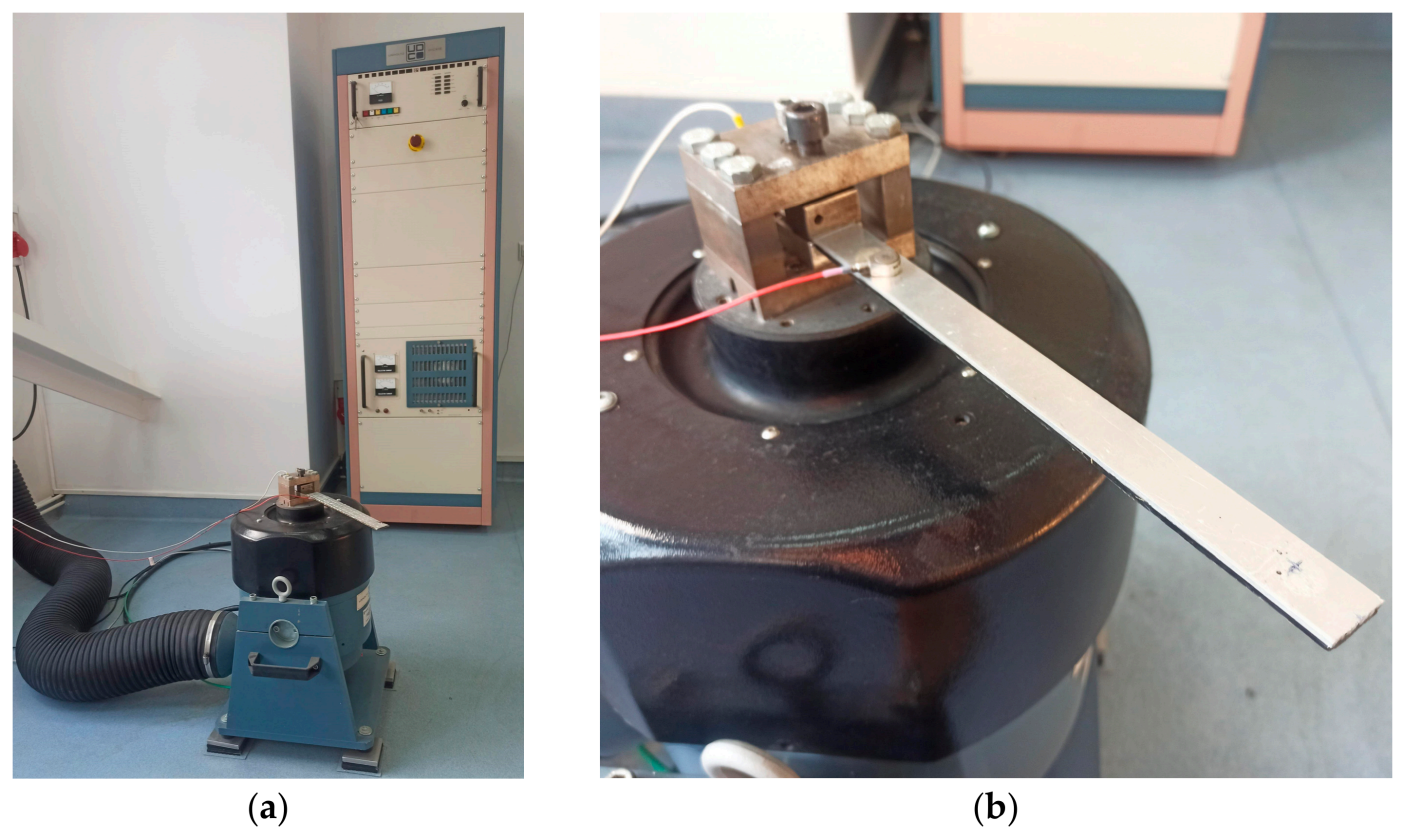
Amplifier and electromagnetic shaker used in experimental investigations (**a**) and the investigated beam fixed to the shaker head (**b**).

Experimental modal tests were carried out using an Unholtz-Dickie UDCO-TA-250 electrodynamic vibration system (Unholtz-Dickie Corporation, European Division, Zuzenhausen, Germany). The test system included an electrodynamic shaker, a power amplifier, a vibration controller, and a computer-based data-acquisition and control system. The investigated cantilever specimens were attached to the moving head of the shaker, as illustrated in Figure 2.

To perform measurements at reduced temperatures, the specimen and the mounting region were enclosed in a thermally insulated chamber developed specifically for the experimental programme. Temperature control was achieved using thermoelectric Peltier modules. The chamber incorporated the thermoelectric assembly, a two-channel temperature controller, a 500 W power supply rated at 12 V and 41.7 A, and a temperature sensor. The enclosure was manufactured from RAVATHERM XPS extruded polystyrene insulation board [22] (Ravago Building Solutions Poland Sp. z o.o., Warsaw, Poland). and was installed directly around the shaker fixture.

**Figure 3:**
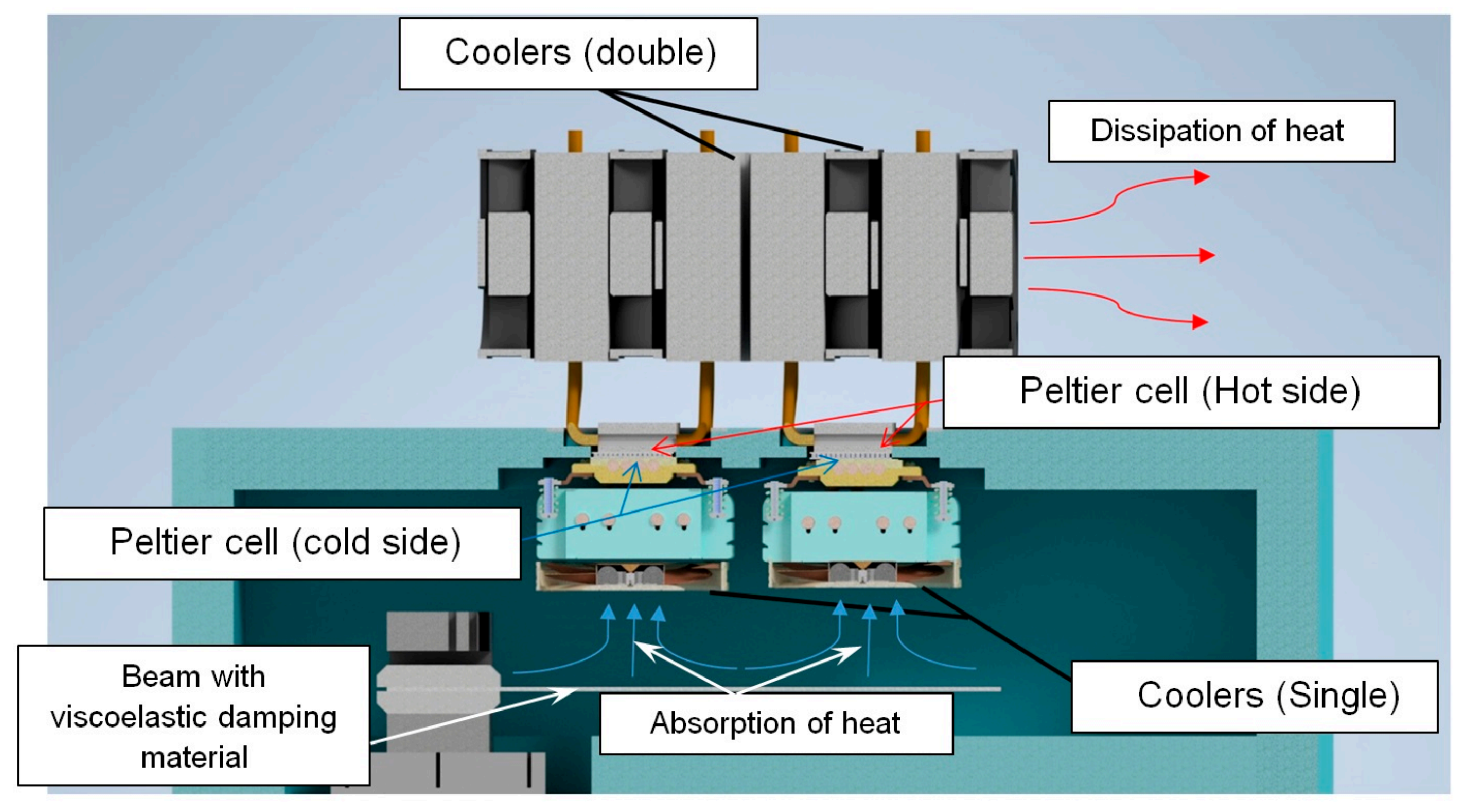
Cross-section of the cooling chamber [21].

The thermoelectric system was arranged so that heat was extracted from the chamber interior and rejected to the surroundings. Each Peltier module was thermally coupled to heat sinks located on both its cold and hot sides. The heat sinks responsible for absorbing heat were positioned inside the chamber, whereas those used for heat rejection were located externally. Thermally conductive paste was applied at the interfaces between the Peltier modules and the heat sinks to improve thermal contact. A schematic cross section of the cooling system is presented in Figure 3.

**Figure 4:**
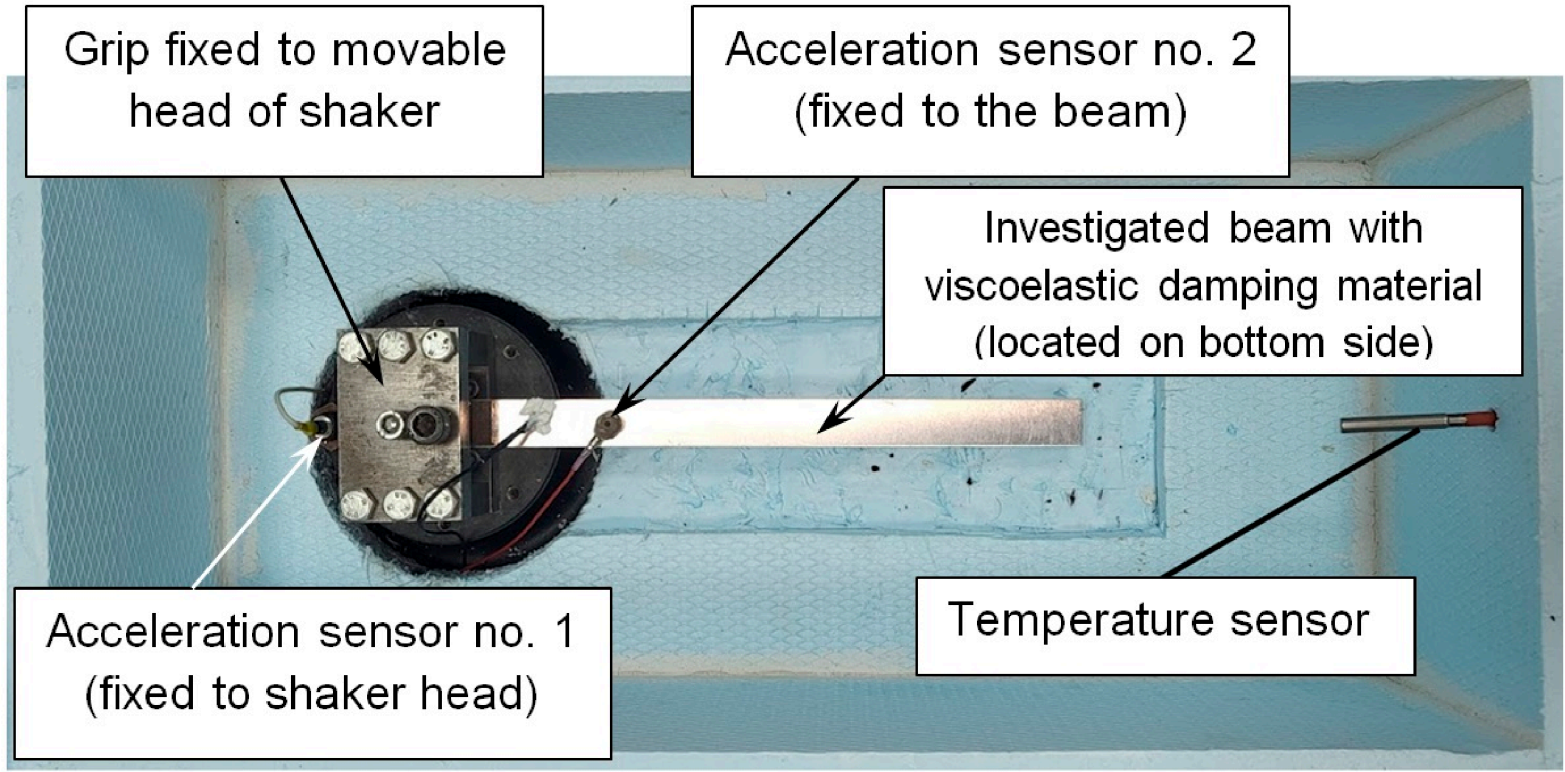
The cooling chamber with visible: grip fixed to shaker head, tested beam, temperature sensor and the piezoelectric acceleration sensors [21].

During testing, the cantilever specimen remained connected to the moving shaker head, while the damping layer was located on the lower surface of the beam. Figure 4 shows the arrangement of the specimen, the shaker fixture, the temperature sensor, and the acceleration sensors inside the chamber.

Vibration measurements were performed using piezoelectric accelerometers [23]. One sensor was mounted on the shaker head and measured the excitation acceleration, denoted as (A_1_). A second accelerometer was attached to the specimen at a distance of 50 mm from the clamped end and measured the beam response, denoted as (A_2_). Endevco 256HX-100 sensors (Endevco Europe, Fribourg, Switzerland) were used. According to the sensor specification, the acceleration measurement uncertainty at a 95% confidence level is ±1.7% in the frequency ranges of 20–120 Hz and 120–2500 Hz, and ±2.7% between 2500 and 4000 Hz.

**Figure 5:**
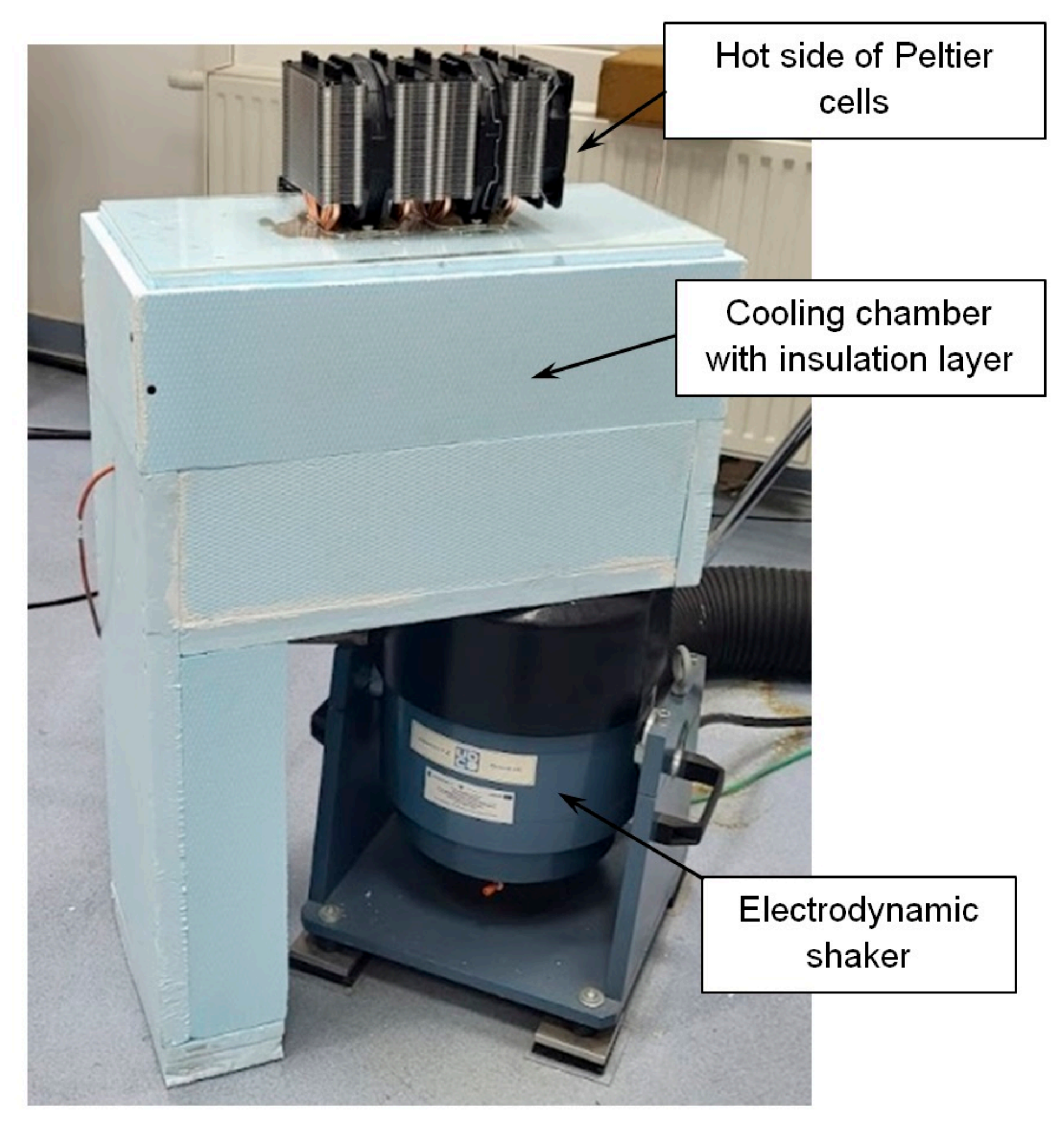
Vibration shaker and cooling chamber (hot side of the Peltier cell is visible on top part of chamber) [21].

The beam was secured in the fixture by means of a clamping mechanism incorporating an M10×1 fine-thread screw. A torque wrench was used during installation to apply the same tightening torque and thereby maintain consistent boundary conditions. After the initial installation, the specimen and accelerometers remained mounted throughout the sequence of measurements performed at different temperatures. This avoided changes in sensor position or clamping conditions between successive tests. The complete shaker and cooling chamber assembly is shown in Figure 5.

Four specimens were investigated. Each specimen was based on a cantilever beam manufactured from AW-2017A aluminium alloy [24,25]. The nominal beam dimensions were 300 mm in length, 20 mm in width, and 1 mm in thickness. Two commercially available viscoelastic materials were examined: a bituminous material and butyl rubber.

**Figure 6:**
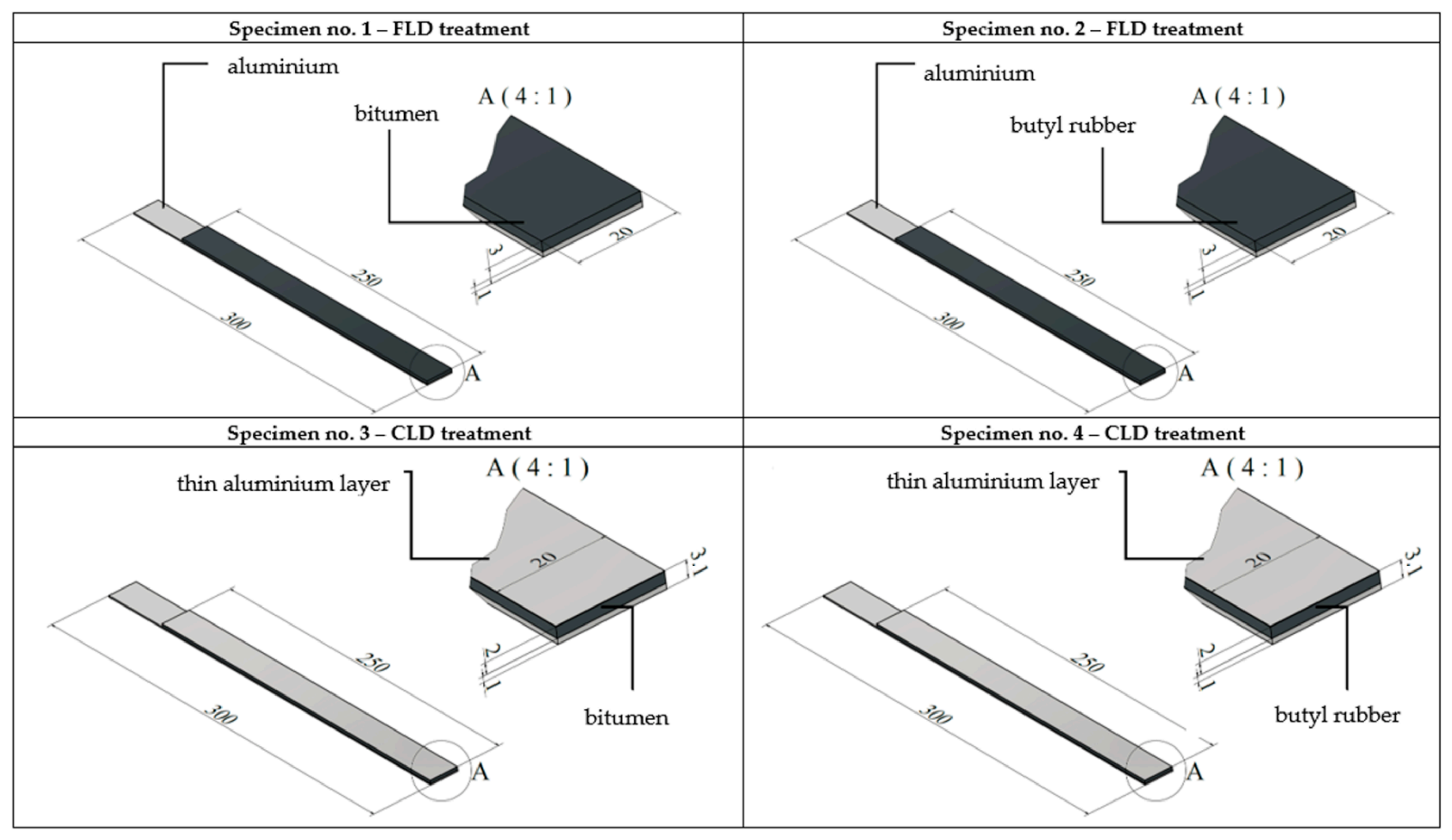
Dimensions and structure of specimens.

For the FLD specimens, a 2 mm thick layer of viscoelastic material was bonded directly to the aluminium beam. Specimen no. 1 incorporated the bituminous material, whereas specimen no. 2 incorporated butyl rubber. The CLD specimens were prepared using the same damping materials but additionally included a 0.1 mm thick aluminium constraining layer bonded to the outer surface of the viscoelastic layer. Consequently, specimen no. 3 represented the CLD configuration with bituminous material, and specimen no. 4 the CLD configuration with butyl rubber. The geometry and layer arrangement of all four specimens are presented in Figure 6.

During specimen preparation, the self-adhesive viscoelastic mats were applied to the aluminium substrate and subsequently rolled to obtain uniform contact over the bonded surface. In the CLD configuration, the additional aluminium constraining layer was attached to the outer surface of the damping material using a polymer adhesive. The densities of the damping materials were approximately 2.0 kg/dm^3^ for butyl rubber and 1.8 kg/dm^3^ for the bituminous material [19].

The specimens were prepared with reference to ASTM E756-05(2017) [26], which provides procedures for evaluating the vibration-damping properties of materials. During each modal test, the shaker was excited by a frequency sweep extending from approximately 40 Hz to 4000 Hz. The sweep frequency increased linearly at a rate of 1 Hz/s.

The acceleration level of the shaker head was maintained at 1 g, where (1 g = 9.81 m/s^2^). The excitation acceleration was recorded by the sensor mounted on the shaker head, while the second accelerometer simultaneously recorded the vibration response of the specimen. The frequency response function was subsequently obtained from the ratio of the measured beam-response amplitude to the shaker-head excitation amplitude.

Measurements were performed at six temperature levels: 22 °C, 18 °C, 15 °C, 10 °C, 5 °C, and −2 °C. The minimum attainable temperature was limited by the cooling capacity of the thermoelectric chamber. The same test sequence was applied to all four specimens, allowing the influence of temperature to be compared directly for the two damping materials and for the FLD and CLD treatment configurations.

## 3. Experimental Results and Discussion

To regulate the air temperature inside the cooling chamber, a temperature controller was used. During the experimental investigation, modal analysis was performed on four specimens (Figure 6) at temperatures of −2 °C, 5 °C, 10 °C, 15 °C, 18 °C, and 22 °C. The lowest temperature investigated was limited by the cooling capacity of the constructed chamber. The first vibration mode was excluded from the analysis due to the large displacement amplitude observed in the beam during this vibration mode.

**Figure 7:**
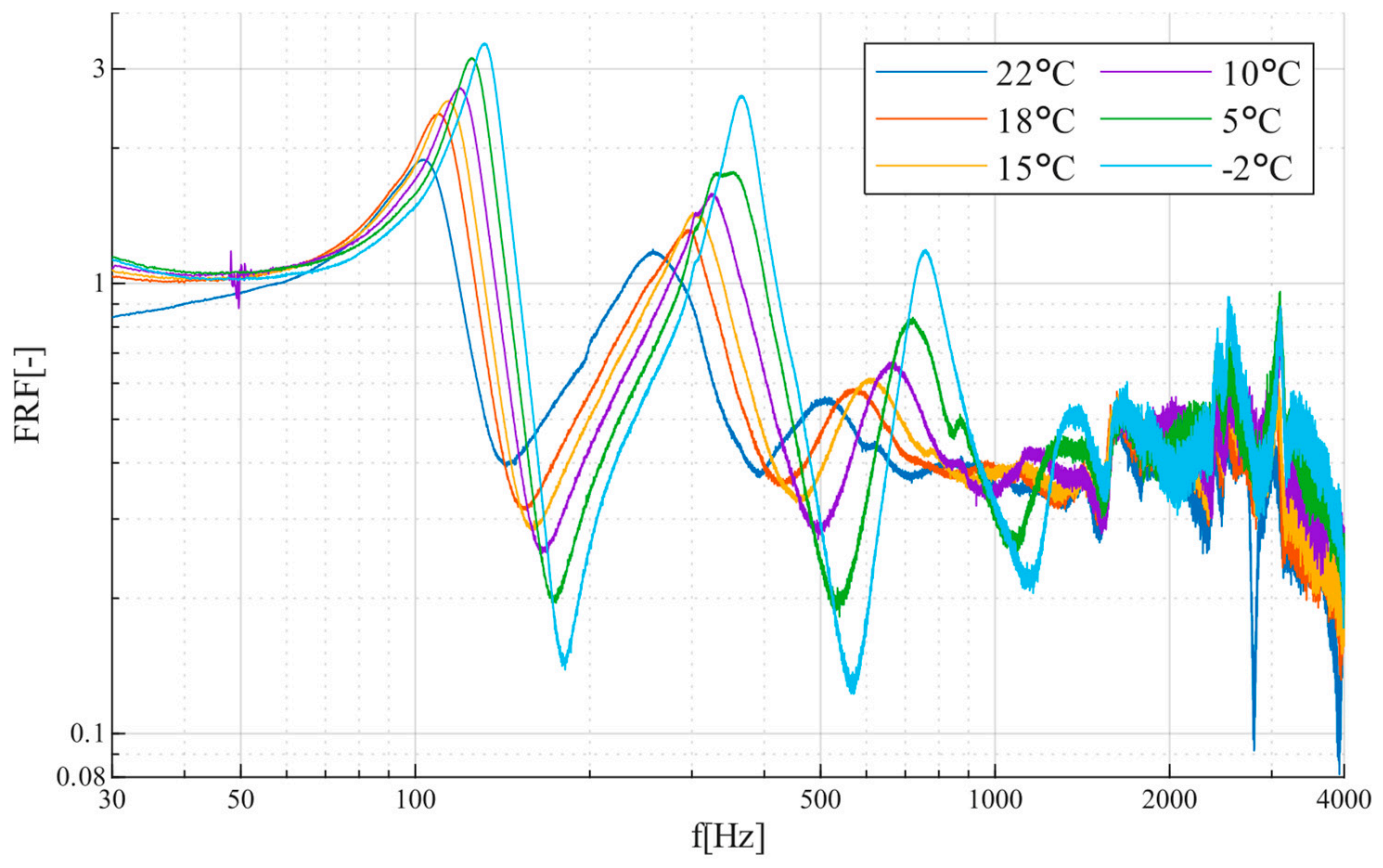
Frequency response curves of the specimen in the FLD configuration, damped with bituminous material (specimen no. 1) at temperatures 22 °C, 18 °C, 15 °C, 10 °C, 5 °C and −2 °C (frequency range: 30–4000 Hz).

**Figure 8:**
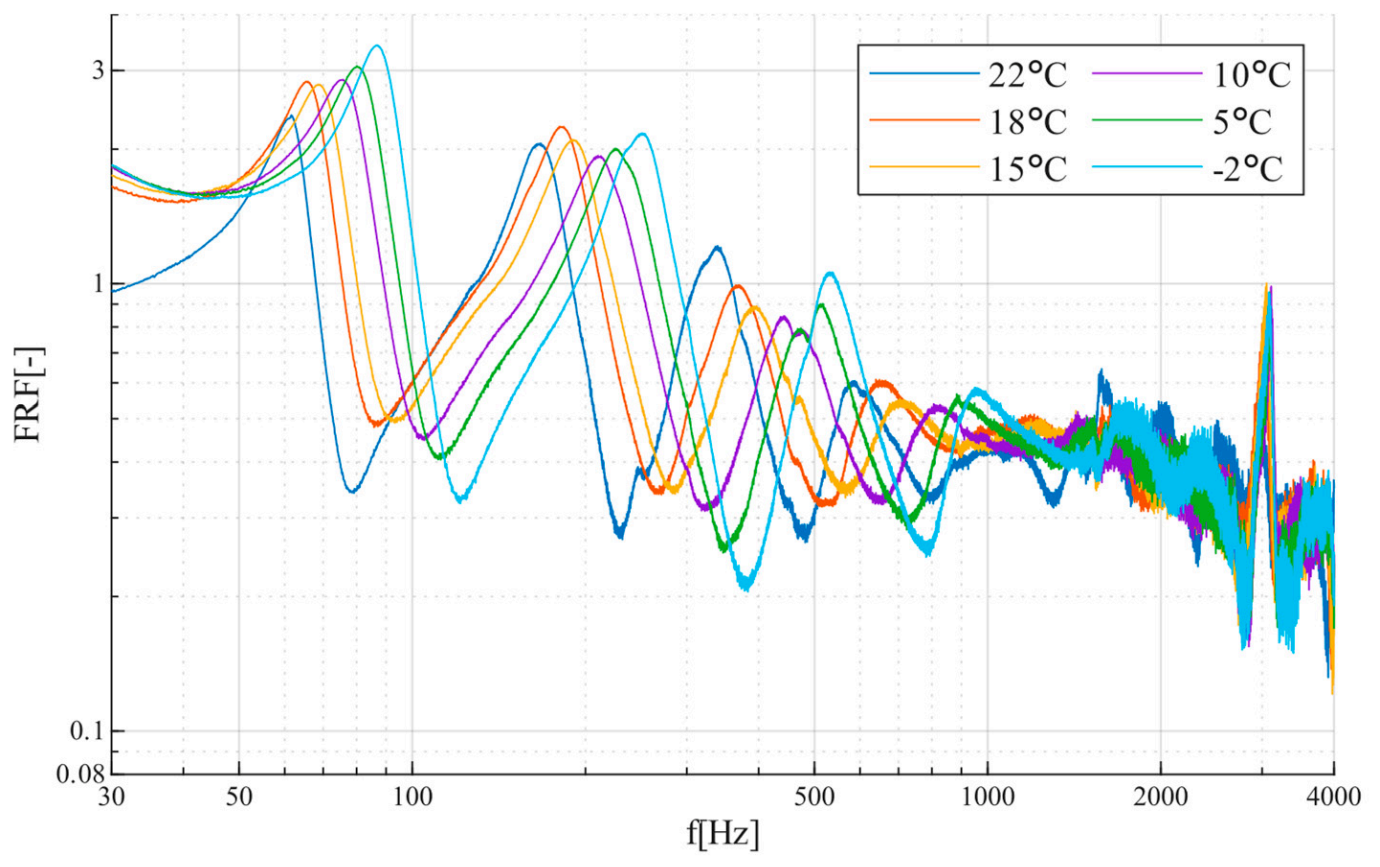
Frequency response curves of the specimen in the CLD configuration, damped with bituminous material (specimen no. 3) at temperatures 22 °C, 18 °C, 15 °C, 10 °C, 5 °C and −2 °C (frequency range: 30–4000 Hz).

The modal response of each specimen was acquired using an LMS measurement system at a frequency resolution of 0.1 Hz. The primary outcome of the experimental study was the set of amplitude-frequency characteristics obtained for the analysed specimens. The vertical axis of the resulting characteristics (Figure 7 and Figure 8) represents the frequency response function (FRF), which describes the system’s response relative to the excitation magnitude. The FRF values were determined by dividing the signal from channel no. 2 (acceleration amplitude A_2_ of the tested specimen) by the signal from channel no. 1 (acceleration amplitude A_1_ measured on the shaker head).

Figure 7 and Figure 8 present the FRFs obtained for the beam specimens damped with the bituminous material using FLD and CLD treatments, respectively, over the investigated temperature range. The recorded FRF curves exhibit distinct local maxima, which were attributed to the resonance responses of the tested specimens. The first peak appearing in the frequency spectrum corresponds to the second bending mode of the beam, as the first mode was omitted from the measurements because the beam could come into contact with the cooling chamber. The most pronounced FRF responses were observed within the first four analysed modes. Above 1 kHz, the differences between the individual FRF curves became negligible. Consequently, the subsequent analysis was restricted to vibration modes 2–5.

The experimental data obtained from the vibration analysis enabled the identification and evaluation of the modal parameters, summarised in Table 1, Table 2, Table 3 and Table 4. The resonance frequencies were denoted as f [Hz], the FRF amplitudes measured at resonance as A [-], and the loss factors as η [-], where the symbol [-] indicates a dimensionless quantity. To distinguish parameters determined for different specimens, the corresponding number value was introduced as a subscript notation. For instance, the parameter f_1_ (Table 1) represents the resonance frequency measured for specimen no. 1. The modal parameters were determined for the second, third, fourth, and fifth vibration modes and subsequently compiled in tabular form. The loss factor values were calculated according to the methodology proposed in Ref. [26].

**Figure 9:**
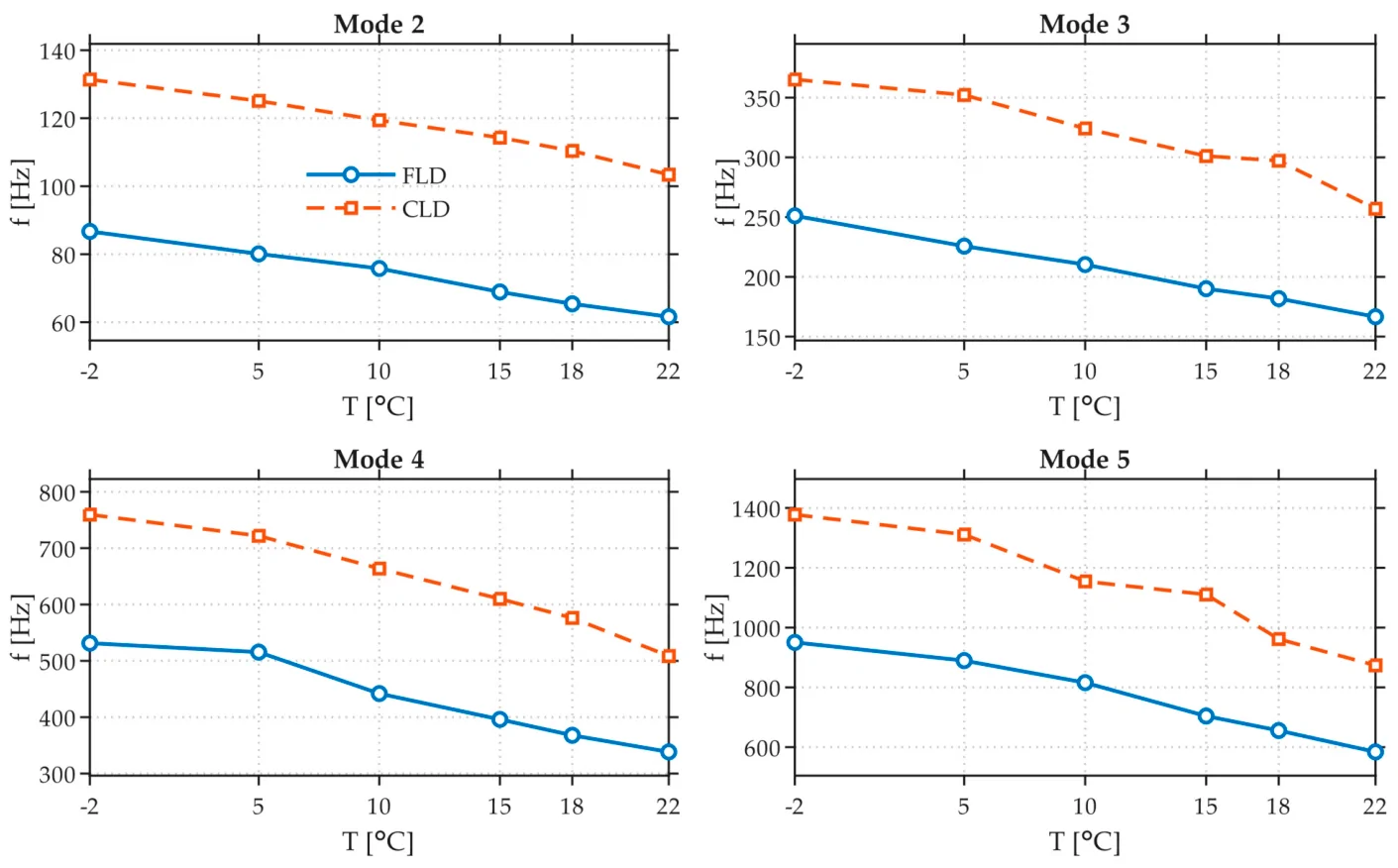
Resonance frequencies of specimens damped with the bituminous material in the FLD (specimen no. 1) and CLD (specimen no. 3) configurations at temperatures ranging from −2 °C to 22 °C. The results are presented for vibration modes 2–5.

**Figure 10:**
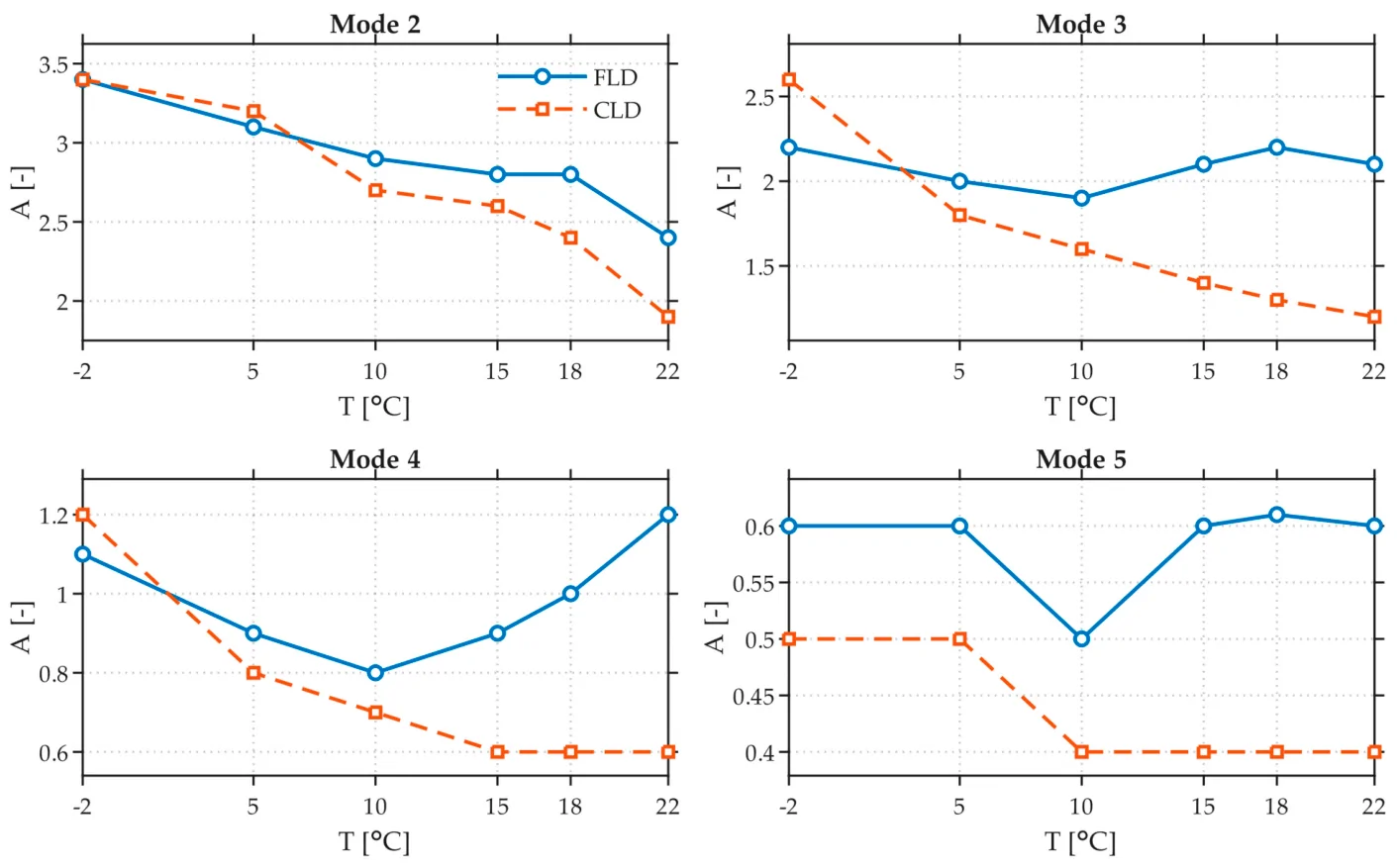
FRF amplitudes at resonance of specimens damped with the bituminous material in the FLD (specimen no. 1) and CLD (specimen no. 3) configurations at temperatures ranging from −2 °C to 22 °C. The results are presented for vibration modes 2–5.

**Figure 11:**
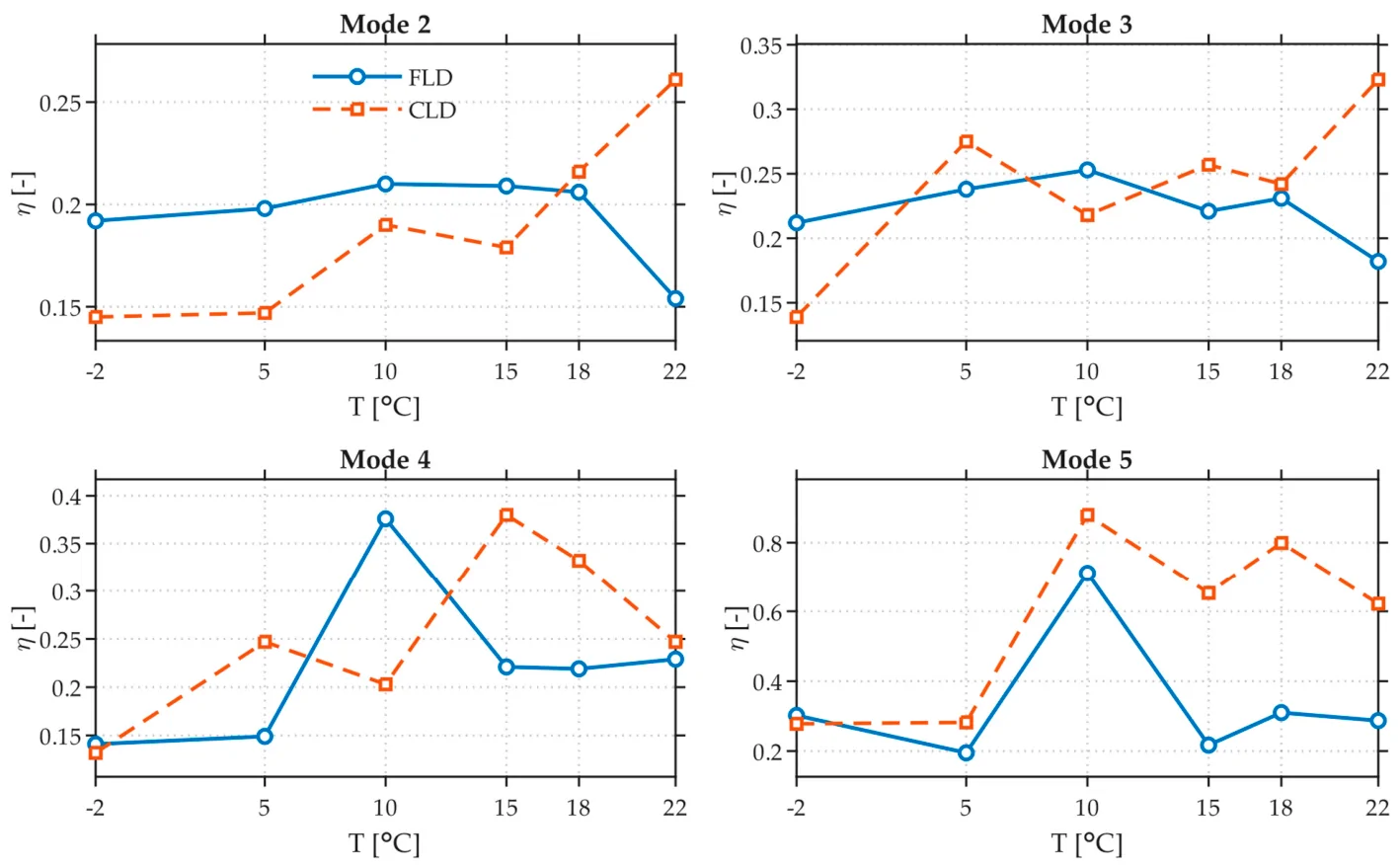
Modal loss factors of specimens damped with the bituminous material in the FLD (specimen no. 1) and CLD (specimen no. 3) configurations at temperatures ranging from −2 °C to 22 °C. The results are presented for vibration modes 2–5.

The results presented in Figure 7, Figure 8, Figure 9, Figure 10 and Figure 11 and Table 1 and Table 2 indicate that lowering the temperature influenced the efficiency of FLD and CLD treatments. At a temperature of 22 °C, the CLD treatment provided better damping. The loss factor η values obtained for modes 2–5 in that case were higher than the loss factor η values measured for the FLD treatment. As the temperature decreased, the damping capabilities of both specimens depended on the mode number and temperature. For instance, at a temperature of 10 °C, specimen no. 1 exhibited higher damping than specimen no. 3 for modes 2–4, but not for mode 5. At the lowest examined temperature (–2 °C, Table 1 and Table 2), the FLD treatment dissipated vibration energy more effectively than the CLD treatment for all examined modes.

**Figure 12:**
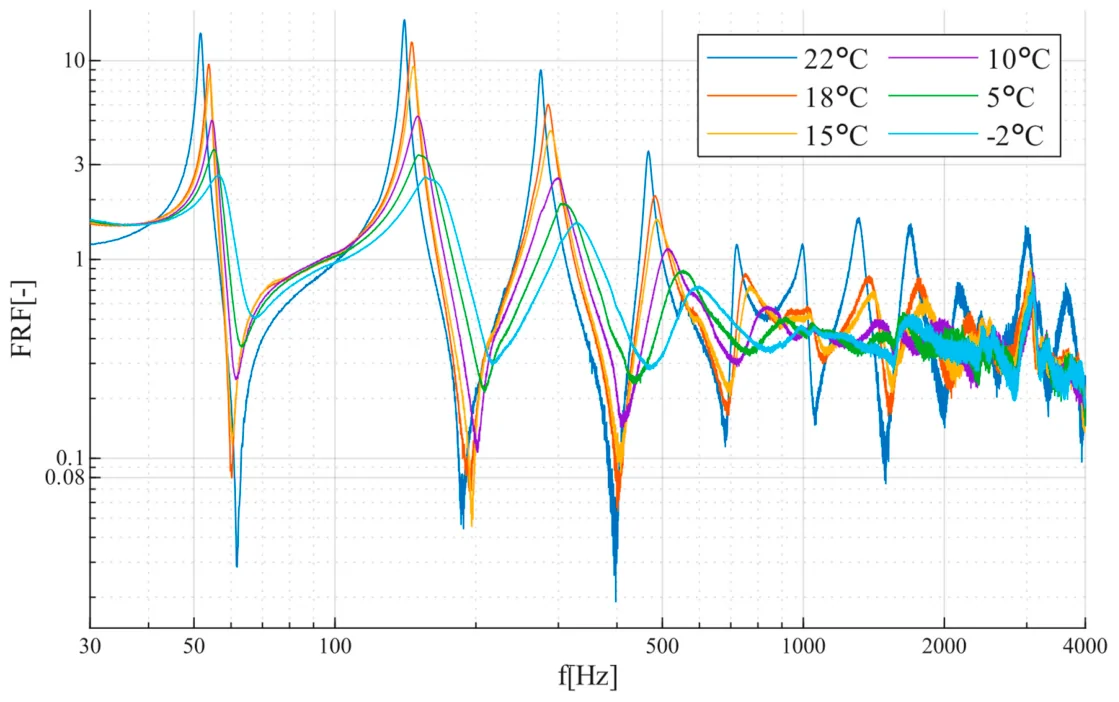
Frequency response curves of specimen in FLD configuration, damped by the butyl rubber (specimen no. 2) at temperatures of 22 °C, 18 °C, 15 °C, 10 °C, 5 °C and −2 °C (frequency range of 30 Hz–4000 Hz).

**Figure 13:**
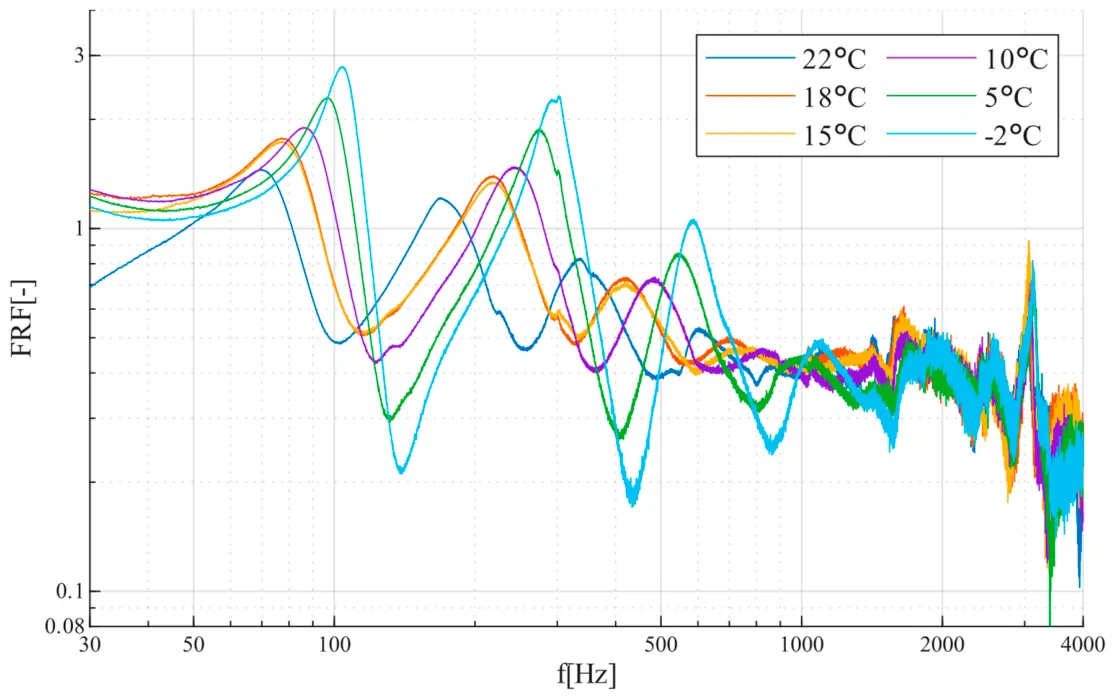
Frequency response curves of specimen in CLD configuration, damped by butyl rubber (specimen no. 4) at temperatures 22 °C, 18 °C, 15 °C, 10 °C, 5 °C and −2 °C (frequency range of 30 Hz–4000 Hz).

Figure 12 and Figure 13 show the FRF of a beam damped with butyl rubber with FLD and CLD treatment. The vertical axis presents FRF values, defined as the ratio of the beam response amplitude to the excitation amplitude. Above 1 kHz, the differences between the individual FRF curves became negligible. Consequently, the subsequent analysis was restricted to vibration modes 2–5.

**Figure 14:**
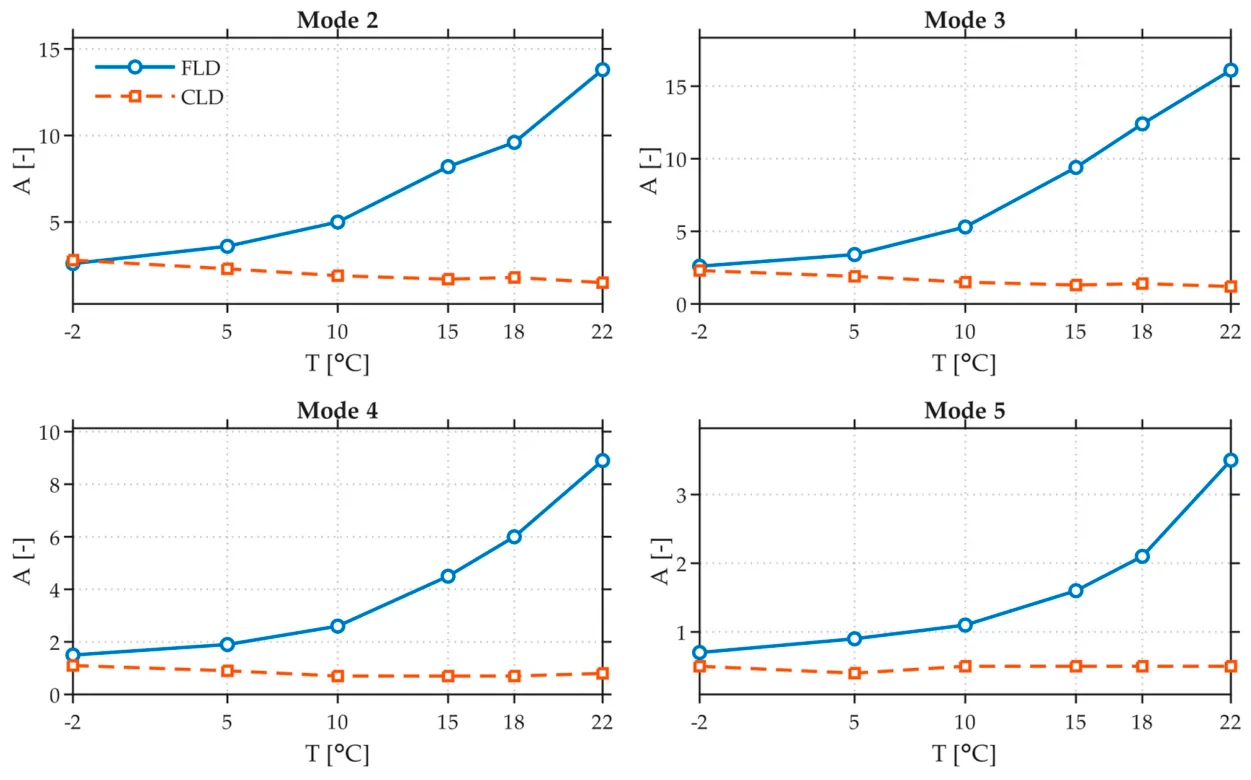
Modal loss factors of specimens damped with the bituminous material in the FLD (specimen no. 2) and CLD (specimen no. 4) configurations at temperatures ranging from −2 °C to 22 °C. The results are presented for vibration modes 2–5.

**Figure 15:**
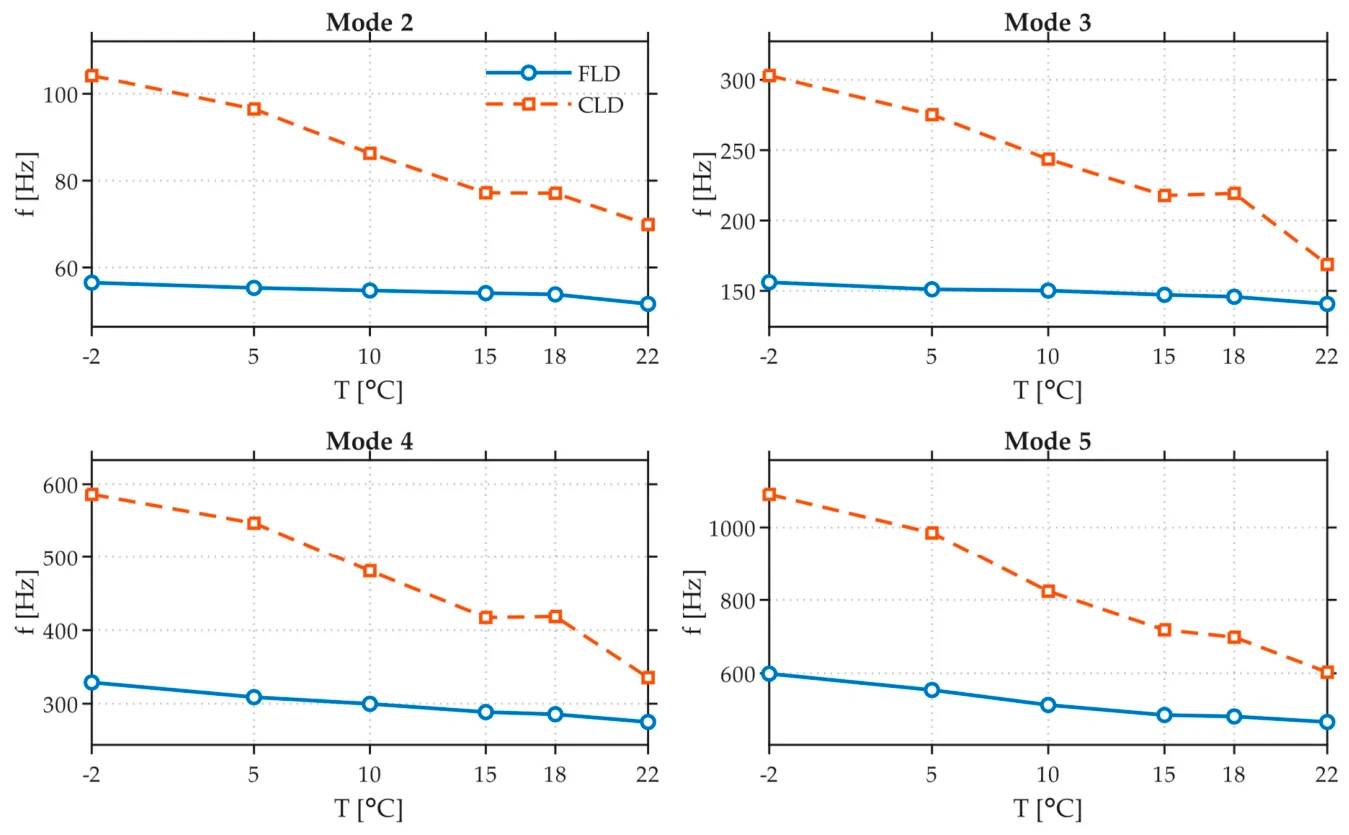
Resonance frequencies of specimens damped with the butyl rubber material in the FLD (specimen no. 2) and CLD (specimen no. 4) configurations at temperatures ranging from −2 °C to 22 °C. The results are presented for vibration modes 2–5.

**Figure 16:**
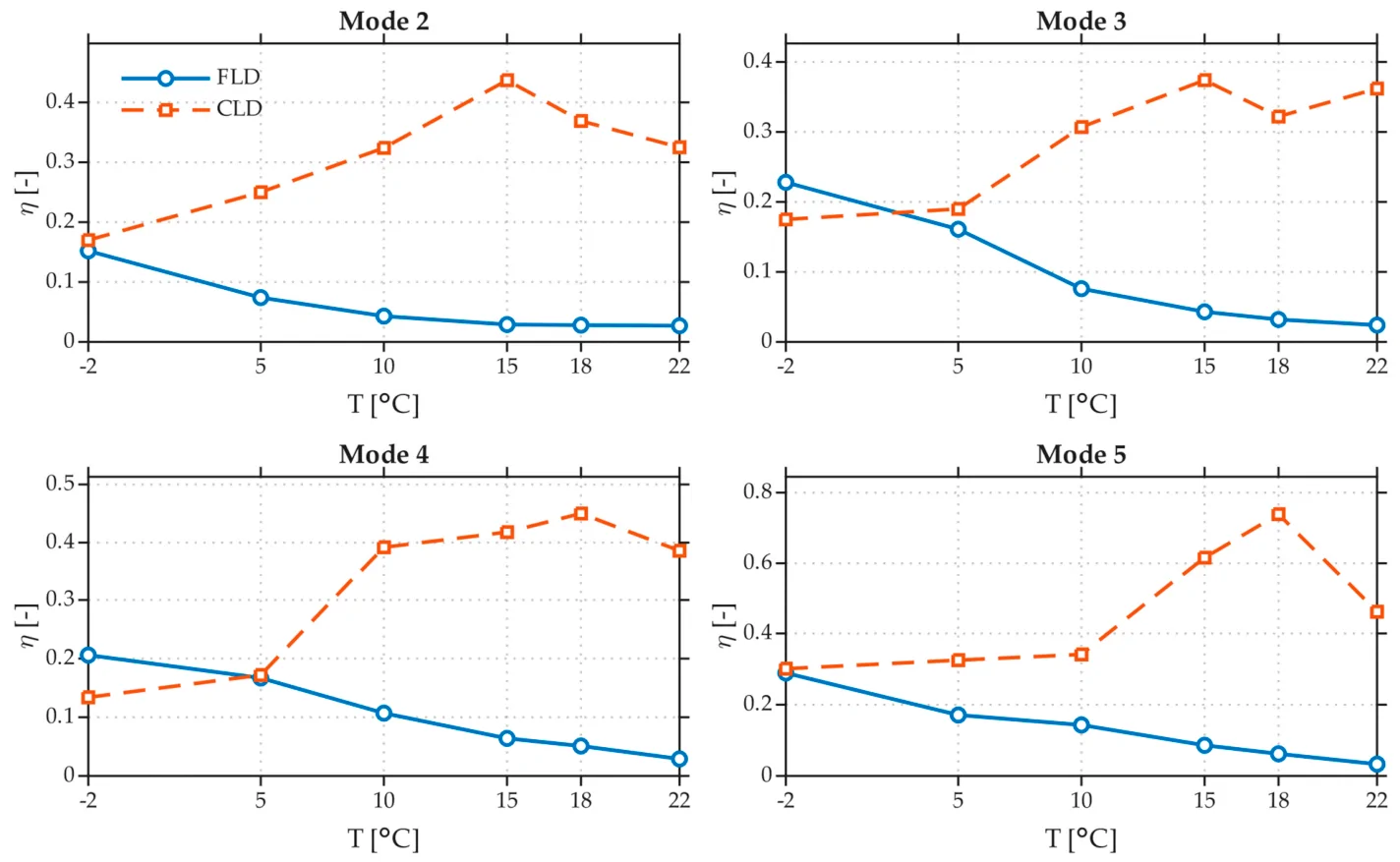
Modal loss factors of specimens damped with the butyl rubber material in the FLD (specimen no. 2) and CLD (specimen no. 4) configurations at temperatures ranging from −2 °C to 22 °C. The results are presented for vibration modes 2–5.

Figure 12 and Figure 13 show that at the highest measured temperature (22 °C), the CLD treatment provided significantly better damping than the FLD treatment. As the temperature decreased, the amplitudes of all modes in specimen no. 2 decreased. It can be seen that at the lowest measured temperature (–2 °C), the amplitudes of modes 2–5 for the FLD treatment were lower than the corresponding amplitudes in specimen no. 4 (CLD). In order to quantitatively compare the modal parameters of the bituminous material, Table 3 and Table 4 and Figure 14, Figure 15 and Figure 16 presents the values of resonant frequency, FRF amplitude at resonance, and loss factor η for the temperature range from –2 °C to 22 °C.

The results presented in Figure 12, Figure 13, Figure 14, Figure 15 and Figure 16 and Table 3 and Table 4 indicate that lowering the temperature influenced the efficiency of FLD and CLD treatments. At a temperature of 22 °C, the CLD treatment provided significantly better damping than the FLD treatment. For modes 2–5, the loss factor η values at this temperature were approximately 10 times lower for specimen no. 2 (FLD). As the temperature decreased, the damping capabilities of both specimens became more similar. For instance, at a temperature of 5 °C, the loss factor η for mode 4 was 0.167 for the FLD treatment and 0.172 for the CLD treatment. At the lowest examined temperature (–2 °C, Table 3 and Table 4), the FLD treatment dissipated vibration energy more effectively than the CLD treatment for modes 3 and 4. In the case of modes 2 and 5, the CLD treatment exhibited slightly higher loss factor values.

The present study is subject to several limitations regarding the investigated temperature and frequency ranges and the lack of repeated measurements. The temperature interval from −2 °C to 22 °C represents a relatively limited portion of the operating conditions that may occur in automotive and aerospace applications and should therefore not be interpreted as a comprehensive representation of their full thermal operating range. The lower temperature limit was determined by the cooling capacity of the experimental chamber used in the present study. Similarly, although the frequency response functions were measured up to 4 kHz, the detailed quantitative analysis was restricted to the second through fifth bending modes. In addition, the lack of repeated measurements prevents a statistical assessment of experimental variability and limits the interpretation of irregular trends observed for some vibration modes. Consequently, the conclusions regarding the relative performance of FLD and CLD treatments should primarily be considered valid within the temperature interval and modal frequency range investigated in this study. Future studies will extend the investigated operating range and include repeated measurements to enable a more comprehensive evaluation of damping performance and a quantitative assessment of experimental variability and the statistical significance of the observed trends.

## 4. Conclusions

The present study investigates the influence of temperature on the vibration-damping performance of viscoelastic materials applied in FLD and CLD configurations. The work focuses on passive vibration damping in lightweight structural elements, with particular relevance to aerospace and automotive applications, where vibration reduction, noise control, and fatigue-life improvement are important design requirements.

Experimental modal analysis was performed on cantilever beams manufactured from AW-2017A aluminium alloy and treated with two viscoelastic damping materials: butyl rubber and a bituminous material. In the FLD configuration, the damping layer was bonded directly to the beam surface, whereas in the CLD configuration the viscoelastic layer was constrained by an additional thin aluminium sheet, promoting shear deformation within the damping layer. The specimens were tested over a temperature range from −2 °C to 22 °C using an electrodynamic shaker, piezoelectric accelerometers, and a thermoelectrically cooled chamber based on Peltier modules. FRFs, resonance frequencies, resonance amplitudes, and modal loss factors were determined for the second to fifth bending modes.

The results demonstrate that temperature has a significant effect on the dynamic stiffness and energy dissipation capacity of the investigated damping treatments. At room temperature, the CLD configuration generally provided superior damping effectiveness, particularly for butyl rubber, where the modal loss factors were substantially higher than those obtained with FLD. As the temperature decreased, the stiffness of the viscoelastic materials increased, resulting in shifts in the natural frequencies and changes in the modal damping performance. For the bituminous material, FLD became more effective than CLD at −2 °C for all analysed vibration modes. In the case of butyl rubber, the difference in damping performance between FLD and CLD gradually decreased with decreasing temperature, and FLD outperformed CLD for selected vibration modes.

The damping performance of FLD and CLD treatments is governed not only by the intrinsic viscoelastic properties of the damping material but also by the deformation mechanism through which mechanical energy is dissipated. Since the mechanical properties of viscoelastic materials are strongly dependent on both temperature and excitation frequency, changes in temperature may significantly alter the effectiveness of a given damping configuration. At higher temperatures, the viscoelastic layer remains relatively compliant, allowing substantial shear deformation to develop between the base structure and the constraining layer. Under these conditions, the CLD configuration is generally more effective because a significant portion of the vibration energy is dissipated through shear deformation within the viscoelastic layer. As the temperature decreases, the storage modulus of the viscoelastic material increases, leading to an increase in its shear stiffness. Consequently, the relative displacement between the constraining and base layers is reduced, resulting in lower shear strains and, therefore, a reduction in the efficiency of the energy dissipation mechanism characteristic of CLD. In contrast, the FLD treatment relies primarily on extensional deformation associated with the bending of the host structure. As the viscoelastic layer becomes stiffer at lower temperatures, it participates more effectively in the bending response and stores a larger portion of the strain energy. Consequently, the difference in damping performance between FLD and CLD gradually decreases.

Overall, the present results confirm that the effectiveness of passive viscoelastic damping treatments depends strongly on both the operating temperature and the vibration mode. The findings indicate that CLD is generally more advantageous under moderate thermal conditions, whereas the effectiveness of FLD increases as the temperature decreases and may equal or even exceed that of CLD, depending on the viscoelastic material and the vibration mode. These observations provide further insight into the temperature-dependent behaviour of FLD and CLD treatments within the investigated range and may contribute to the selection of passive damping solutions for automotive and aerospace structures. However, extrapolation of the present results beyond the investigated temperature and modal frequency ranges requires further experimental verification.

## Figures and Tables

**Table 1:** Frequency of resonant vibration, amplitude of FRF during resonance and loss factor η for beam damped by the bituminous material (specimen no. 1 (FLD)) at temperatures 22 °C, 18 °C, 15 °C, 10 °C, 5 °C and −2 °C.

Temp.	FLD Mode 2			FLD Mode 3			FLD Mode 4			FLD Mode 5		
	$f_{1}$ [Hz]	$A_{1}$ [-]	$\eta_{1}$ [-]	$f_{1}$ [Hz]	$A_{1}$ [-]	$\eta_{1}$ [-]	$f_{1}$ [Hz]	$A_{1}$ [-]	$\eta_{1}$ [-]	$f_{1}$ [Hz]	$A_{1}$ [-]	$\eta_{1}$ [-]
**22 °C**	61.6	2.4	0.154	166.6	2.1	0.182	338.4	1.2	0.229	584.1	0.6	0.287
**18 °C**	65.4	2.8	0.206	181.8	2.2	0.231	367.9	1	0.219	655.6	0.61	0.31
**15 °C**	68.9	2.8	0.209	190.1	2.1	0.221	396.1	0.9	0.221	704.1	0.6	0.217
**10 °C**	75.8	2.9	0.21	210.3	1.9	0.253	441.9	0.8	0.376	815.7	0.5	0.711
**5 °C**	80.1	3.1	0.198	225.7	2	0.238	515.5	0.9	0.149	815.7	0.5	0.711
**−2 °C**	86.7	3.4	0.192	251.1	2.2	0.212	531.4	1.1	0.141	950.4	0.6	0.302

**Table 2:** Frequency of resonant vibration, amplitude of FRF during resonance and loss factor η for beam damped by the bituminous material (specimen no. 3 (CLD)) at temperatures 22 °C, 18 °C, 15 °C, 10 °C, 5 °C and −2 °C.

Temp.	CLD Mode 2			CLD Mode 3			CLD Mode 4			CLD Mode 5		
	$f_{3}$ [Hz]	$A_{3}$ [-]	$\eta_{3}$ [-]	$f_{3}$ [Hz]	$A_{3}$ [-]	$\eta_{3}$ [-]	$f_{3}$ Hz	$A_{3}$ [-]	$\eta_{3}$ [-]	$f_{3}$ [Hz]	$A_{3}$ [-]	$\eta_{3}$ [-]
**22 °C**	103.4	1.9	0.261	257	1.2	0.323	508.5	0.6	0.247	873.5	0.4	0.622
**18 °C**	110.4	2.4	0.216	297.2	1.3	0.242	576.2	0.6	0.332	961.6	0.4	0.799
**15 °C**	114.3	2.6	0.179	301.1	1.4	0.257	610	0.6	0.38	1110.5	0.4	0.653
**10 °C**	119.4	2.7	0.19	324.2	1.6	0.218	663.7	0.7	0.203	1154.5	0.4	0.88
**5 °C**	125.1	3.2	0.147	352.2	1.8	0.275	721.9	0.8	0.247	1311.2	0.5	0.282
**−2 °C**	131.4	3.4	0.145	365.2	2.6	0.139	759.5	1.2	0.132	1377.8	0.5	0.278

**Table 3:** Frequency of resonant vibration, amplitude of FRF during resonance, and loss factor η for beam damped by butyl rubber (specimen no. 2 (FLD)) at temperatures 22 °C, 18 °C, 15 °C, 10 °C, 5 °C and −2 °C.

Temp.	FLD Mode 2			FLD Mode 3			FLD Mode 4			FLD Mode 5		
	$f_{2}$ [Hz]	$A_{2}$ [-]	$\eta_{2}$ [-]	$f_{2}$ [Hz]	$A_{2}$ [-]	$\eta_{2}$ [-]	$f_{2}$ [Hz]	$A_{2}$ [-]	$\eta_{2}$ [-]	$f_{2}$ [Hz]	$A_{2}$ [-]	$\eta_{2}$ [-]
**22 °C**	51.6	13.8	0.027	140.7	16.1	0.024	275.1	8.9	0.029	467.2	3.5	0.033
**18 °C**	53.8	9.6	0.028	145.8	12.4	0.032	285.6	6	0.051	482.3	2.1	0.062
**15 °C**	54.1	8.2	0.029	147.2	9.4	0.043	288.6	4.5	0.064	486.2	1.6	0.086
**10 °C**	54.7	5	0.043	150.2	5.3	0.076	299.8	2.6	0.107	513.6	1.1	0.143
**5 °C**	55.3	3.6	0.074	151.1	3.4	0.161	308.9	1.9	0.167	554.4	0.9	0.171
**−2 °C**	56.5	2.6	0.152	156.1	2.6	0.228	328.9	1.5	0.206	598.9	0.7	0.289

**Table 4:** Frequency of resonant vibration, amplitude of FRF during resonance, and loss factor η for beam damped by butyl rubber (specimen no. 4 (CLD)) at temperatures of 22 °C, 18 °C, 15 °C, 10 °C, 5 °C and −2 °C.

Temp.	CLD Mode 2			CLD Mode 3			CLD Mode 4			CLD Mode 5		
	$f_{4}$ [Hz]	$A_{4}$ [-]	$\eta_{4}$ [-]	$f_{4}$ [Hz]	$A_{4}$ [-]	$\eta_{4}$ [-]	$f_{4}$ [Hz]	$A_{4}$ [-]	$\eta_{4}$ [-]	$f_{4}$ [Hz]	$A_{4}$ [-]	$\eta_{4}$ [-]
**22 °C**	69.9	1.5	0.325	168.8	1.2	0.362	335.5	0.8	0.386	602.9	0.5	0.461
**18 °C**	77.1	1.8	0.369	219.3	1.4	0.322	418.6	0.7	0.45	698.4	0.5	0.739
**15 °C**	77.2	1.7	0.437	217.8	1.3	0.374	417.3	0.7	0.418	718.4	0.5	0.616
**10 °C**	86.3	1.9	0.324	243.6	1.5	0.307	481	0.7	0.392	823.6	0.5	0.341
**5 °C**	96.5	2.3	0.25	275.2	1.9	0.19	546.9	0.9	0.172	985.1	0.4	0.325
**−2 °C**	104.2	2.8	0.17	303	2.3	0.175	586.2	1.1	0.134	1090.5	0.5	0.301

## Data Availability

The original contributions presented in the study are included in the article, further inquiries can be directed to the corresponding author.
